# Cervical Cancer Screening and Human Papillomavirus Vaccination among Korean Sexual Minority Women by Sex of Their Sexual Partners

**DOI:** 10.3390/ijerph17238924

**Published:** 2020-11-30

**Authors:** Ssirai Kim, Sun-Young Lee, Smi Choi-Kwon

**Affiliations:** 1 College of Nursing, Seoul National University, Seoul 03080, Korea; ssirai@snu.ac.kr; 2 Department of Healthcare Administration, Seoul National University Hospital, Seoul 03080, Korea; 3 Public Healthcare Center, Seoul National University Hospital, Seoul 03080, Korea; sy2376@gmail.com; 4 The Research Institute of Nursing Science, Seoul National University, Seoul 03080, Korea

**Keywords:** cervical cancer, sexual minority women, cancer prevention, South Korea

## Abstract

Cervical cancer-preventive behaviors in Korean sexual minority women (SMW) are underexplored. We aimed to assess the differences in cervical cancer screening uptake and completion of human papillomavirus (HPV) vaccination among Korean SMW by sex of their sexual partners. This cross-sectional study used data from the 2017 Korean Sexual Minority Women’s Health Study; we included Korean lesbian and bisexual women aged ≥20 years. They were divided into three groups: SMW with more than one male sexual partner (male only/both), SMW with only female sexual partners (female-only), or no sexual partner (no partner). Among the 671 participants, 266 (39.6%), 294 (43.8%), and 111 (16.5%) belonged to the male-only/both, female-only, and no partner groups, respectively. Compared to the male-only/both group, the female-only group was significantly less likely to have undergone cervical cancer screening (Adjusted odds ratio (AOR) = 0.24, 95% confidence interval (CI) = 0.15–0.37) and to have completed HPV vaccinations (AOR = 0.58, 95% CI = 0.37–0.91). In conclusion, Korean SMW with only female sexual partners had lower cervical cancer screening and HPV vaccination completion rates than SMW who had male sexual partners. More extensive efforts are needed to improve cervical cancer-preventive behaviors among Korean SMW.

## 1. Introduction

Although cervical cancer is preventable through regular cervical screening or human papilloma virus (HPV) vaccination, it remains a major public health problem worldwide [1]. The Centers for Disease Control and Prevention in the United States (US) recommends that all women over the age of 20 years should be screened for cervical cancer regardless of their sexual history [2]. The Advisory Committee on Immunization Practices routinely recommends HPV vaccination at a young age [3]. However, previous studies have reported that sexual minority women (SMW) are less likely to undergo cervical cancer screening than the general female population [4,5]. In addition, another study has reported lower rates of HPV vaccination among SMW [6]. This low cervical cancer screening and low HPV vaccination rates among SMW may be partly due to misconceptions [7,8]; these include the notions that SMW are less susceptible to cervical cancer and sex with women is safer than sex with men [8]. Lower utilization of cervical cancer screening tests among lesbians has been associated with lower perceived cancer risk [7,9]. Marrazzo et al., however, reported that there were cervical neoplasia cases even in women who reported never having sex with men [10]. In addition, HPV transmission was reported to be possible through female-to-female sexual activities [11]. Although cervical cancer screening is necessary regardless of the sex of sexual partners, SMW showed different behaviors according to the sex of the sexual partners. In a previous study on the screening rate of cervical cancer according to the sexual partners of young women in the US, cervical cancer screening rates were lower in women who had sexual experiences only with women than in those who had sexual experiences only with men [5,12]. Therefore, to understand the sexual health of SMW and lower their risk of cervical cancer, it is important to evaluate cervical cancer-preventive behaviors according to the sex of the sexual partners of SMW [4,13].

The incidence of cervical cancer remains higher in South Korea than in other countries [14,15], despite the provision of free cervical cancer screening services [16]. The age-standardized incidence rates of cervical cancer per 100,000 people are 8.4, 6.5, 5.7, and 6.7 in Korea, the USA, Canada, and France, respectively [17]. In Korea, cervical cancer screening through Papanicolaou smears (PAP smears) is provided bi-annually free of cost to women aged over 20 years [16]. Vaccination is also recommended for women aged 25–26 years or younger who have not completed the vaccination course before. The HPV vaccination has been provided free of charge to girls aged 12 years since 2016 [18]. Since SMW in Korea have been reported to experience various health inequalities, including healthcare utilization, compared to the general female population [19,20], it is likely that they also have lower cervical cancer screening and HPV vaccination rates. In recent studies, Korean SMW showed negative health results because of discrimination and reported more risky health behaviors than the general population [19,21]. However, preventive healthcare utilization for cervical cancer among young SMW has yet to be investigated. Moreover, according to previous studies, cervical cancer-preventive behaviors vary depending on the sex of the sexual partners of SMW [4,5,12]. No studies have examined cervical cancer screening and HPV vaccination rates according to the sex of sexual partners of Korean SMW.

Thus, the purpose of this study was to investigate the cervical cancer screening and HPV vaccination rates of Korean SMW according to the sex of their sexual partners. We also investigated the reasons why the study participants did not undergo cervical cancer screening and vaccinations against HPV.

## 2. Materials and Methods

### 2.1. Study Design and Participants

This cross-sectional study used data from the Korean Sexual Minority Women’s Health Study, which was an online survey designed to assess the health behaviors and quality of life of sexual minority women in Korea. The data were collected between September 2017 and November 2017. The participants were Koreans aged 19 years or older and self-identified as lesbians, bisexual women, or other sexual minorities. Gender minorities, such as trans men and trans women, were not included in the survey. The participants were recruited through social media platforms such as Twitter and Facebook, LGBT-targeted electronic newsletters, or LGBT online communities. All participants were informed about the study’s purpose and design through an online post. Online questionnaires were allowed to be completed only once in a single device to prevent duplication of responses; among the 1256 respondents, 801 completed the survey.

**Figure 1:**
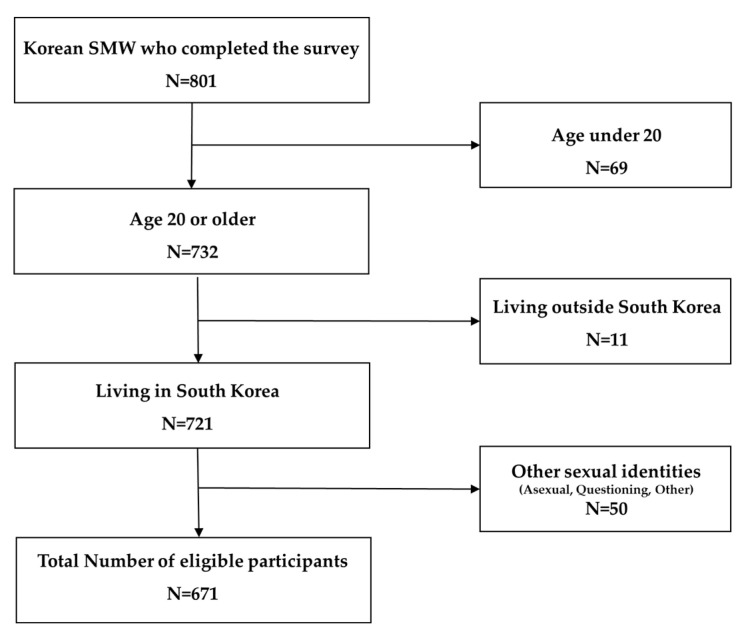
Flow diagram describing the selection of eligible participants.

In the current study, we excluded participants under the recommended age for cervical cancer screening, as well as SMW living outside Korea because their healthcare access would differ from that of Korean residents. In total, 721 SMW living in Korea who were aged ≥20 years were initially selected. Among them, we further excluded 50 participants who answered “asexual”, “questioning”, or “other” for the sexual identity question. Finally, 671 women were included in our analysis (Figure 1).

### 2.2. Study Variables

#### 2.2.1. Sexual Identity and Sex of Sexual Partners

In the original survey, for the question regarding their sexual identity, participants could choose among lesbian, bisexual, heterosexual, pansexual, asexual, and questioning categories, or even directly input their sexual identity. In the current study, we included lesbians, bisexuals, and pansexuals and divided them into two groups for the analysis, namely, lesbians and bisexuals; pansexuals were included in the bisexual group.

We investigated whether the participants had had sexual experiences with men or women throughout their life. They were accordingly divided into three groups: male-only/both, those with more than one male sexual partner; female-only, those with only a female sexual partner without a male partner; and no partner, those who did not have either a male or female sexual partner.

#### 2.2.2. Cervical Cancer Screening and HPV Vaccination Completion

Cervical cancer screening status within the past 2 years was set as the primary outcome measure. The year of the last screening was investigated, and screening included a PAP smear test and liquid-based cytology. In the analysis of cervical cancer screening in the past 2 years, only participants whose final screening took place between 2016 and 2017 were included.

HPV vaccination status was set as the secondary outcome. Vaccine completion was defined as having completed the HPV vaccine series.

#### 2.2.3. Medical History

Previous studies found that the history of hospital utilization, such as sexually transmitted infection (STI) service use [12,22], influenced cervical cancer screening. Thus, we included STI treatment experience and breast cancer screening experience from the raw data. STI treatment experience was defined as having a history of hospital treatment for STIs. Meanwhile, the breast cancer screening experience was defined as having undergone breast cancer screening in the past year.

#### 2.2.4. Reasons for Being Unscreened or Unvaccinated

Participants were asked for reasons for having never been screened for cervical cancer and having never received the HPV vaccination. Multiple answers were allowed for each question. The reasons for the non-screening of cervical cancer were as follows: (1) “I do not think I am the subject of the screening”, (2) “There was no time for the screening”, (3) “There was no interest in the screening”, (4) “It was because of the cost”, (5) ”I am not having sex”, (6) “I did not know about the screening”, (7) ”I think I am healthy”, (8) ”I have sex only with women”, (9) “I am afraid that the medical staff will know that I am a sexual minority”, (10) “I think the screening will be painful”, (11) “Screening would be embarrassing”, and (12) ”I have a fear of a negative result”. Meanwhile, the reasons for non-vaccination against HPV were as follows: (1) “I do not think I am the subject of the vaccination”, (2) “There was no time for vaccination”, (3) “There was no interest in the vaccination”, (4) “It was because of the cost”, (5) “I am not having sex”, (6) “I did not know about the vaccination”, (7) “I think I am healthy”, (8) “I have sex only with women”, (9) “I am afraid that the medical staff will know that I am a sexual minority”, (10) “I think the injection will be painful”, (11) “The effect of the vaccination is doubtful”, and (12) “I am worried about side effects”.

#### 2.2.5. Sociodemographic Variables

Sociodemographic characteristics included age, education level, income level, job, and presence of a current partner. Age was divided into five categories: 20–24, 25–29, 30–34, 35–39, and ≥40 years. Educational level was divided into two categories: high school graduate or under and college student/graduate or higher. Annual household income (based on the average income per household in Korea as of 2016, 1 USD to approximately 1000 KRW [23]) was categorized into <$50,000 and ≥$50,000. Job-status was identified as student, employed, or unemployed. The presence of a current partner was classified as yes or no.

### 2.3. Statistical Analysis

Participants’ general characteristics were compared based on the sex of their sexual partners. The frequency and percentage of the participants’ general characteristics, sexual identity, cervical cancer screening, HPV vaccination status, and reasons for not undergoing cervical cancer screening and HPV vaccination were calculated according to the sex of the sexual partners. Categorical variables are presented as counts and proportions and were compared using the chi-squared test. Continuous variables are presented as medians and inter-quartile ranges. Logistic regression analysis was used to estimate the adjusted odds ratio (AOR) and 95% confidence interval (CI) for the association between the study outcomes and the sex of the sexual partners. For each outcome, after fitting the bivariate models, we adjusted for age (Model 1). We then adjusted the model by adding education level, income level, and job status (Model 2). Finally, we adjusted the model by including sexual identity, the presence of a current partner, and medical history (Model 3). All statistical analyses were performed using SAS software, version 9.4 (SAS Institute Inc., Cary, NC, USA). *p*-values were based on a two-sided significance level of 0.05.

### 2.4. Ethics Declarations

We obtained informed consent from the survey participants through an online survey form, and the study was approved by the institutional review board of the Seoul National University (IRB No. 1707/002-005).

## 3. Results

### 3.1. Sociodemographic Characteristics by Sex of Sexual Partner

The median age of participants was 25 years, 91.8% had an education level of college graduate or above, 29.1% earned an above-average Korean household income, and 47.4% were employed. Overall, 52.8% had a current partner, and 52.9% were lesbians. Meanwhile, 39.6% have had more than one male sexual partner in the past, and 43.8% had only female partners. Among SMW with past male sexual partners, 32.3% were self-identified lesbians. The participants’ characteristics are shown in Table 1.

The rate of cervical cancer screening within the last 2 years was 22.4%. With respect to the screening rate by group, the rates were 38.3% and 14.3% in the male-only/both and female-only groups, respectively. Meanwhile, the HPV vaccination completion rate was 31.2% for the male-only/both group, 20.4% for the female-only group, and 24.3% for the no partner group.

### 3.2. Logistic Regression Analysis of Cervical Cancer Screening

After adjustment for socioeconomic factors (Model 2), the female-only and no partner groups were less likely to have undergone cervical cancer screening than the male-only/both group (AOR = 0.24, 95% CI = 0.15–0.37 and AOR = 0.14, 95% CI = 0.06–0.34, respectively). In the fully adjusted model (Model 3), the odds of cervical cancer screening within 2 years were still lower in the female-only and no partner groups than in the male-only/both group (AOR = 0.32, 95% CI = 0.19–0.52 and AOR = 0.23, 95% CI = 0.09–0.59, respectively) (Table 2).

### 3.3. Logistic Regression Analysis of HPV Vaccination

After adjustment for socioeconomic factors (Model 2), the female-only group was less likely to have completed the HPV vaccine series than the male-only/both group (AOR = 0.53, 95% CI = 0.36–0.79). In the fully adjusted model (Model 3), the HPV vaccination completion rate was still lower for the female-only group (AOR = 0.58, 95% CI = 0.37–0.91) than for the male-only/both group. In Model 3, participants with a high annual income were more likely to have completed the HPV vaccine series (AOR = 1.98, 95% CI = 1.35–2.90) (Table 3).

### 3.4. Reasons for Being Unscreened for Cervical Cancer or Unvaccinated against HPV

Among the participants who had never undergone cervical cancer screening, the primary reason was no interest in the screening test, accounting for 37.1% of all the answers. The second most common reason for not undergoing screening was only having sex with women, with 29.3% of the respondents citing this reason. Meanwhile, the primary reason for never availing of the HPV vaccination was the cost, with 34.9% of the respondents having reported this reason (Table 4).

## 4. Discussion

In this study, the rate of cervical cancer screening in the past 2 years among Korean SMW was 22.4%. We found a higher rate of cervical cancer screening in the past two years in Korean SMW who had sex with more than one male partner in their lifetime than in those who never had sex with a male partner. The HPV vaccination completion rate among Korean SMW in our study was 25.3%. The vaccination rates were also similarly higher in Korean SMW who had male sexual partners than in those who only had female sexual partners.

Research on cervical cancer-preventive behaviors among Korean SMW is scarce. To the best of our knowledge, this is the first study to examine the cervical cancer screening and HPV vaccination rates among SMW in Korea, especially with respect to the sex of their sexual partners. In this study, the rate of cervical cancer screening among Korean SMW in the last two years was lower than that of the general population or that of SMW in western countries, which was reported to be between 43% and 69% [7,12,24]. Our findings are consistent with those of previous reports, which showed that the screening rate of SMW was lower than that of the general population [5,25]. There may be several reasons for the low cervical cancer screening rates among SMW in Korea. First, the screening rate of the general Korean female population was also lower than that of populations in other countries [26]. The national cervical cancer screening rate in the Korean female population was only 54.4% [27]. It is not common for Korean women to visit obstetricians, especially if they are not married [28,29]; this may have influenced the screening rate of SMW. Second, the low screening rate may have been due to the lack of accurate information. Although cervical cancer screening is provided free of charge in Korea, more than one-fourth (26.2%) of our unscreened participants answered that they did not receive the screening due to the cost. Previous studies revealed that cervical cancer screening rates were high when SMW disclosed their sexual orientations to the medical staff [22,30]. It may therefore be inferred that the low cervical cancer screening rate among Korean SMW was among other reasons, it is difficult for SMW to disclose their sexual orientations to others due to Korean culture. However, in our study, only 8.5% of the participants selected “I am afraid medical staff will know that I am a sexual minority” as the reason for being unscreened, although multiple choices were allowed.

We found that the rate of cervical cancer screening in our participants differed according to the sex of their sexual partners. The screening rate for cervical cancer was lower in SMW who had sex only with female partners than in those who had sex with one or more male partners. In addition, those who had no past sexual partner received fewer examinations than the male-only/both group. Our results were partially in agreement with those of previous studies [4,12]. A previous study showed that women who had female sexual partners had a lower screening rate for cervical cancer than those with only male partners [4]. In another study, SMW with female sex partners were less likely to be screened for cervical cancer than those with male sex partners [12].

Our findings indicated that sexual experience with a male partner was an essential factor influencing the cervical cancer screening rate among Korean SMW. The lower screening rate in the female-only group could be due to misconceptions about cervical cancer screening. This was supported by our finding that 49.6% of the women who were never screened answered that they did not undergo regular screening because they had sex only with women. Misconceptions due to lack knowledge were indicated as barriers to screening in previous studies [7,30]. Adequate knowledge regarding screening recommendations and perceived benefits of screening was positively associated with cervical cancer screening [7,8,30]. The reasons cited by non-screeners echoed the importance of SMW-specific cervical cancer screening information. Future studies should directly assess the knowledge related to cervical cancer in Korean SMW.

The HPV vaccination completion rate in our study (25.3%) was lower than that in those conducted in the US, which ranged from 32% to 36% [4,31,32]. However, both previous and current studies demonstrated low HPV vaccination rates [31,32]. This may be partially attributed to the age of provision of free national HPV vaccination. HPV vaccinations have been provided free of charge to girls aged 12 years in Korea since 2016. Meanwhile, people outside this target population would need to bear the expenses of the vaccination. However, HPV vaccination is recommended for women aged 25–26 years or younger [18]. The cancer-preventive effect has not been proven in women aged ≥27 years; however, women aged ≥27 years with no sexual history or those who are less likely to be exposed to HPV can theoretically benefit from the vaccination [3,18].

The HPV vaccination completion rate among Korean SMW varied according to the sex of their sexual partners. The HPV vaccination rate was lower in the female-only group than in the male-only/both group. Interestingly, there was no significant difference in the HPV vaccination rate between the male-only/both group and the no partner group. Among the unvaccinated participants in the female-only group, 47.3% reported that they did not avail of the vaccination because they only had sex with women. Nearly half (48.9%) of the unvaccinated participants in the no partner group reported they did not receive the vaccine because they never had sex. However, the HPV vaccination is recommended from a young age, regardless of sexual experience [1]. This suggested that SMW lack knowledge regarding the HPV vaccination. A previous study found that the perceived benefits of the HPV vaccination and perceived susceptibility to HPV infections predicted the attitudes and intentions to avail the HPV vaccine in young Korean women [33]. Further investigation is needed on the health-related beliefs about the HPV vaccination in Korean SMW. With respect to the HPV vaccination rates according to income, it is notable that, similar to previous studies [31,34], the rates in this study differed according to the annual household income. In contrast, there was no difference in the cervical cancer screening rates according to annual household income in the current study, which could primarily be attributed to the fact that the government funds cervical cancer screening. Economic status is an important factor for vaccinations without state cost subsidies. The HPV vaccination rate itself was not high, highlighting the need for further government funding to reduce vaccine cost.

Our findings suggest that specific guidance is needed for SMW when providing the national examination program. There is a need for promoting the benefits that SMW may obtain from regular cervical cancer screening; this will lower the disparity in cervical cancer screening between the general population and SMW in Korea. In addition, healthcare professionals need to use customized education programs and approaches based on the sex of sexual partners to reduce differences in cervical cancer screening rates and HPV vaccinations among SMW.

This study had some limitations. First, we only used convenience sampling to recruit participants because data on sexual identity were not collected in the national questionnaire. This sampling process limits the generalizability of our findings. Second, an online platform was used to reach more SMW in Korea. This could have resulted in the inclusion of a higher proportion of younger participants in our study. In the future, national-level studies covering a more comprehensive age range are needed. Third, data on cervical cancer screening and HPV vaccination were self-reported and were not confirmed by medical records. Fourth, causality could not be inferred because we used self-reported answers for the reasons for being unscreened and unvaccinated to interpret the related factors. Despite these limitations, we believe that this study is valuable because, to the best of our best knowledge, it is the first to examine the factors associated with cervical cancer screening and HPV vaccine completion among SMW in Korea.

## 5. Conclusions

The cervical cancer screening and HPV vaccination rates among Korean SMW were lower than those reported in previous studies. The screening and vaccination rates differed according to the sex of the past sexual partner among SMW in Korea. Those who had male sexual partners had higher cervical cancer screening rates in the past two years and were more likely to be vaccinated against HPV than those who had sex only with female partners. These findings indicated that including the sex of sexual partners in the baseline sexual history assessment in clinical practice would help in understanding the behaviors related to cervical cancer screening and HPV vaccination among Korean SMW. In addition, further efforts are needed to adequately inform this population regarding cervical cancer-preventive behaviors and their benefits, particularly in those who have sexual experiences only with women.

## Figures and Tables

**Table 1:** Descriptive statistics by sex of sexual partners.

	Total		Sex of Sexual Partner ^1^						*p*-Value
			Male Only/Both		Female Only		No Partner		
	*N*	%	*N*	%	*N*	%	*N*	%	
Total	671	100.0	266	100.0	294	100.0	111	100.0	
Age group									<0.001
20–24	302	45.0	97	36.5	117	39.8	88	79.3	
25–29	194	28.9	90	33.8	89	30.3	15	13.5	
30–34	100	14.9	49	18.4	47	16.0	4	3.6	
35–39	44	6.6	16	6.0	24	8.2	4	3.6	
40–51	31	4.6	14	5.3	17	5.8	0	0.0	
Median (Q1–Q3)	25 (22–30)		26 (23–30)		26 (23–30)		22 (21–24)		
Education level									0.623
High school or under	55	8.2	25	9.4	21	7.1	9	8.1	
Above college	616	91.8	241	90.6	273	92.9	102	91.9	
Annual income									0.265
<$50,000	476	70.9	198	74.4	201	68.4	77	69.4	
≥$50,000	195	29.1	68	25.6	93	31.6	34	30.6	
Job									<0.001
Student	293	43.7	95	35.7	125	42.5	73	65.8	
Employed	318	47.4	144	54.1	143	48.6	31	27.9	
Unemployed	60	8.9	27	10.2	26	8.8	7	6.3	
Current partner									<0.001
Yes	354	52.8	162	60.9	182	61.9	10	9.0	
Sexual identity									<0.001
Lesbian	355	52.9	86	32.3	228	77.6	41	36.9	
Bisexual	316	47.1	180	67.7	66	22.4	70	63.1	
Medical history									
STI ^2^ treatment	50	7.5	44	16.5	5	1.7	1	0.9	<0.001
Breast exam	118	17.6	60	22.6	47	16.0	11	9.9	0.008
Cervical cancer screening									<0.001
in the last 2 years	150	22.4	102	38.3	42	14.3	6	5.4	
HPV vaccination									0.013
Yes	170	25.3	83	31.2	60	20.4	27	24.3	

^1^ Male only/both: SMW with more than one male sexual partner; female-only: SMW with only female sexual partners; no partner: SMW with no past sexual partner; ^2^ STI: sexually transmitted infections.

**Table 2:** Multivariable logistic regression analysis of cervical cancer screening by sex of sexual partners.

		Total	Outcome		Unadjusted			Adjusted ^1^								
								Model 1			Model 2			Model 3		
			*N*	%	OR	95% CI		AOR	95% CI		AOR	95% CI		AOR	95% CI	
Cervical cancer screening in 2 years																
Total		671	150	22.4												
Partner ^2^	Male only/both	266	102	38.3	1.00			1.00			1.00			1.00		
	Female-only	294	42	14.3	0.27	0.18	0.40	0.24	0.16	0.37	0.24	0.15	0.37	0.32	0.19	0.52
	No partner	111	6	5.4	0.09	0.04	0.22	0.14	0.06	0.35	0.14	0.06	0.34	0.23	0.09	0.59

^1^ Model 1: adjusted for age; model 2: adjusted for age, job, education level, and income level; model 3: adjusted for age, job, education level, income level, sexual identity, current partner, STI treatment, and breast exam; ^2^ male-only/both: SMW with more than one male sexual partner; female-only: SMW with only female sexual partners; no partner: SMW with no past sexual partner.

**Table 3:** Multivariable logistic regression analysis of human papillomavirus (HPV) vaccination by sex of sexual partners and socioeconomic status.

		Total	Outcome		Unadjusted			Adjusted ^1^								
								Model 1			Model 2			Model 3		
			*N*	%	OR	95% CI		AOR	95% CI		AOR	95% CI		AOR	95% CI	
HPV vaccination																
Total		671	170	25.3												
Partner ^2^	Male only/both	266	83	31.2	1.00			1.00			1.00			1.00		
	Female-only	294	60	20.4	0.57	0.39	0.83	0.57	0.39	0.84	0.53	0.36	0.79	0.58	0.37	0.91
	No partner	111	27	24.3	0.71	0.43	1.18	0.71	0.42	1.21	0.67	0.39	1.15	0.72	0.40	1.29
Socioeconomic status																
	High education level	616	161	26.1										1.63	0.75	3.54
	High annual income	195	67	34.4										1.98	1.35	2.90
	Employed (ref. student)	318	80	25.2										1.02	0.62	1.68
	Unemployed (ref. student)	60	15	25.0										0.99	0.47	2.05

^1^ Model 1: adjusted for age; model 2: adjusted for age, job, education level, and income level; model 3: adjusted for age, job, education level, income level, sexual identity, current partner, STI treatment, and breast exam; ^2^ male-only/both: SMW with more than one male sexual partner; female-only: SMW with only female sexual partners; no partner: SMW with no past sexual partner.

**Table 4:** Reasons for non-screening of cervical cancer and non-vaccination against HPV (*n* = 451) ^1^.

	Total		Sex of Sexual Partner ^2^					
			Male Only/Both		Female Only		No Partner	
	*N*	%	*N*	%	*N*	%	*N*	%
**Reasons for non-screening of cervical cancer in a lifetime**								
Total	451	100.0	127	100	224	100.0	100	100.0
I do not think I am the subject of the screening	73	16.2	16	12.6	32	14.3	25	25.0
There was no time for the screening	141	31.3	54	42.5	58	25.9	29	29.0
There was no interest for the screening	167	37.1	50	39.4	86	38.4	31	31.0
It was because of the cost	118	26.2	56	44.1	42	18.8	20	20.0
I am not having sex	87	19.3	5	3.9	24	10.8	58	58.0
I did not know about the screening	39	8.7	9	7.1	18	8.1	12	12.0
I think I am healthy	97	21.6	34	26.8	47	21	16	16.0
I have sex only with women	132	29.3	17	13.4	111	49.6	4	4.0
I’m afraid that the medical staff will know I’m a sexual minority	38	8.5	4	3.1	34	15.2	0	0.0
I think the screening will be painful	39	8.7	15	11.8	18	8.1	6	6.0
Screening would be embarrassing	52	11.6	17	13.4	23	10.3	12	12.0
I have a fear of the negative result	23	5.1	15	11.8	7	3.2	1	1.0
**Reasons for non-vaccination against HPV**								
Total	493	100.0	176	100	233	100.0	84	100.0
I don’t think I’m the subject of the vaccination	46	9.4	13	7.4	21	9.1	12	14.3
There was no time for the vaccination	119	24.2	46	26.1	50	21.5	23	27.4
There was no interest in the vaccination	162	32.9	55	31.3	79	34	28	33.4
It was because of the cost	172	34.9	86	48.9	62	26.7	24	28.6
I am not having sex	65	13.2	3	1.7	21	9.1	41	48.9
I did not know about vaccination	62	12.6	24	13.6	21	9.1	17	20.3
I think I am healthy	88	17.9	32	18.2	43	18.5	13	15.5
I have sex only with women	142	28.9	29	16.5	110	47.3	3	3.6
I’m afraid that the medical staff will know I’m a sexual minority	16	3.3	4	2.3	12	5.2	0	0
I think the injection will be painful	32	6.5	14	8	13	5.6	5	6
The effect of the vaccination is doubtful	75	15.3	37	21	29	12.5	9	10.8
I am worried about side effects	110	22.4	52	29.5	45	19.4	13	15.5

^1^ Multiple responses were allowed; ^2^ male-only/both: SMW with more than one male sexual partner; female-only: SMW with only female sexual partners; no partner: SMW with no past sexual partner.
